# Case report: Fibrotic interstitial lung disease as the initial manifestation of hereditary pulmonary alveolar proteinosis caused by CSF2RB mutation

**DOI:** 10.3389/fphar.2023.1252193

**Published:** 2024-01-08

**Authors:** Qiuhong Li, Huikang Xie, Manhui Li, Kebin Cheng

**Affiliations:** ^1^ Department of Respiratory and Critical Care Medicine, Shanghai Pulmonary Hospital, School of Medicine, Tongji University, Shanghai, China; ^2^ Department of Pathology, Shanghai Pulmonary Hospital, School of Medicine, Tongji University, Shanghai, China

**Keywords:** CSF2RB, fibrotic interstitial lung disease, chest high-resolution computed tomography, mutation, pulmonary alveolar proteinosis

## Abstract

A 50-year-old male was admitted to the hospital with a 3-year history of dyspnea and cough. Chest high-resolution computed tomography (HRCT) did not show typical features of pulmonary alveolar proteinosis (PAP), but rather atypical features of interstitial lung disease with fibrosis. The diagnosis of PAP was confirmed through transbronchial lung cryobiopsy. Whole exome sequencing identified a rare homozygous frame shift mutation (c.304_305del:p.S102Ffs*5) in exon 3 of the CSF2RB gene in our patient. This case represents a rare occurrence of fibrotic interstitial lung disease in PAP.

## Introduction

Pulmonary alveolar proteinosis (PAP) is a syndrome characterized by surfactant accumulation in the alveoli, partly due to disruption of GM-CSF signaling. GM-CSF cell-surface receptors on alveolar macrophages are composed of alpha and beta chains encoded by CSF2RA and CSF2RB. Hereditary PAP (herPAP) is caused by CSF2RA or CSF2RB mutations (Trapnell et al., 2019), which block GM-CSF binding to the membrane and prevent GM-CSF signals. This disruption impairs surfactant catabolism, leading to PAP. Mutations of CSF2RA and CSF2RB commonly cause intra-alveolar surfactant accumulation without interstitial infiltration (Borie et al., 2011). CSF2RB mutations have been reported to cause pediatric hereditary PAP but rarely cause herPAP in older adults (Tanaka et al., 2011). In this paper, we describe a case of an adult herPAP patient with an uncommon presentation of fibrotic interstitial lung disease caused by CSF2RB mutation.

## Case report

A 50-year-old male was admitted to the hospital with a 3-year history of dyspnea and cough. The patient occasionally experiences fever and produces sputum. He has never smoked or consumed alcohol. Apart from a surgical history of traumatic brain injury, the patient has no other significant medical history. He worked as a carpenter for over 20 years but left the occupation 10 years ago. During his time as a carpenter, he was exposed to wood dust and decoration pollution.

The patient’s weight was 77.2 kg, with a height of 168 cm, and body mass index of 27.35 kg/m^2^. Physical examination revealed decreased breath sounds with crackles in both lower lung fields. Spirometry results showed a forced expiratory volume in 1 s (FEV1) of 2.44 L (76.2% of predicted), forced vital capacity (FVC) of 2.71 L (69.5% of predicted), and FEV1/FVC ratio of 89.7%. The pulmonary diffusion function was severely reduced, with a diffusion capacity of carbon monoxide (DLco) at 34% of predicted. All serum auto-antibodies tested were negative and the GM-CSF auto-antibody test was negative.

**FIGURE 1:**
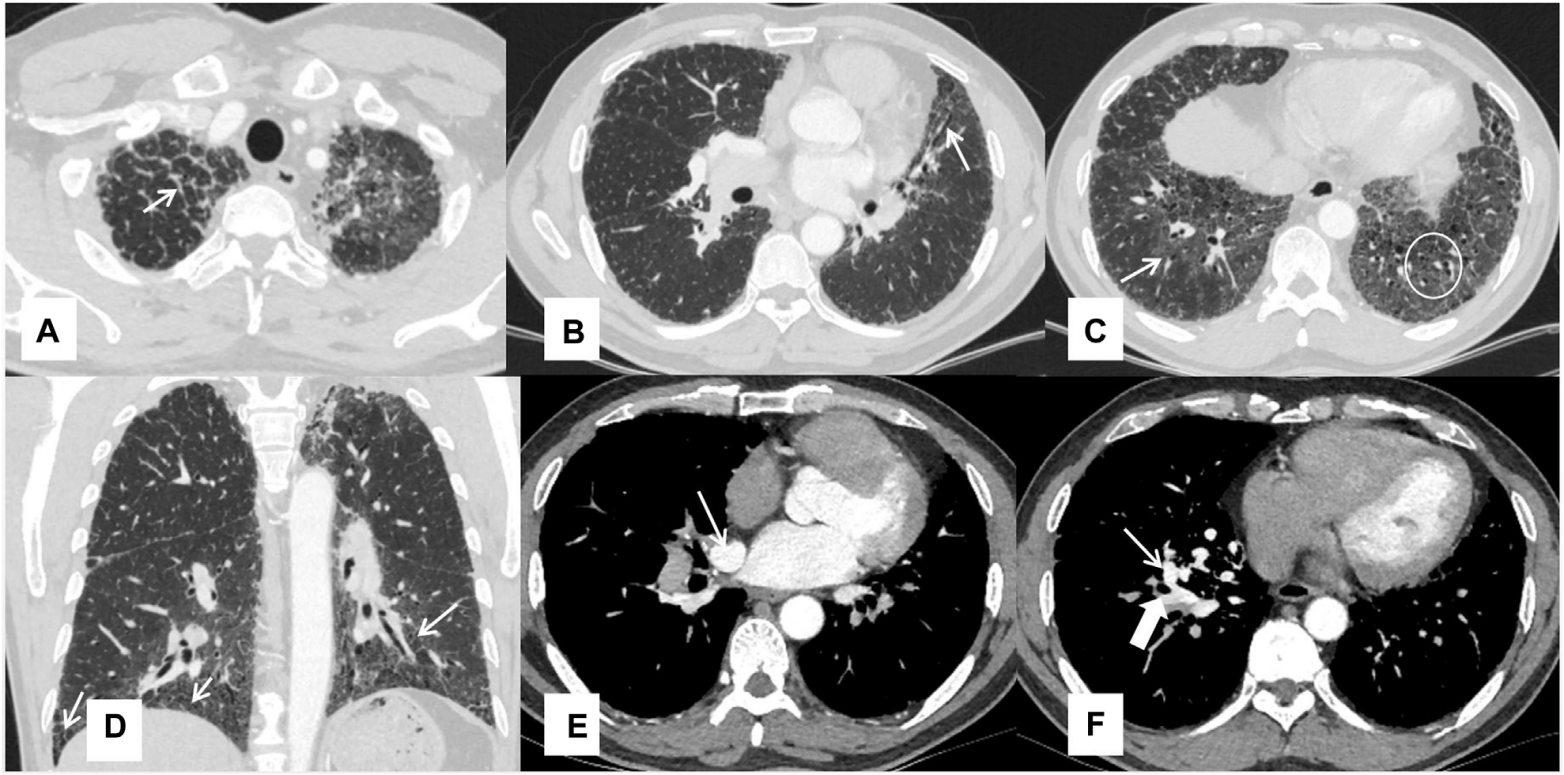
Chest HRCT features of the patient Interlobular septal thickening **(A)**, traction bronchiectasis **(B)**, ground-glass opacities pointed by a thin arrow and reticular shadows in a white circle **(C)**, reticular shadows **(D)**, and the right upper inferior vein (thin arrow) and right inferior pulmonary vein (thick arrow) **(E, F)**.

Chest HRCT imaging did not show the typical crazy-paving pattern, but features of fibrosis were observed, such as peri-bronchovascular and interlobular septal thickening, reticular abnormality, architectural distortion, and traction bronchiectasis. Some ground-glass opacities were also observed. Computed tomography angiography demonstrated an abnormal communication between the right upper pulmonary vein and the right inferior pulmonary vein at the right hilum, due to the absence of connection between the right superior pulmonary vein and the left atrium (Figure 1).

**FIGURE 2:**
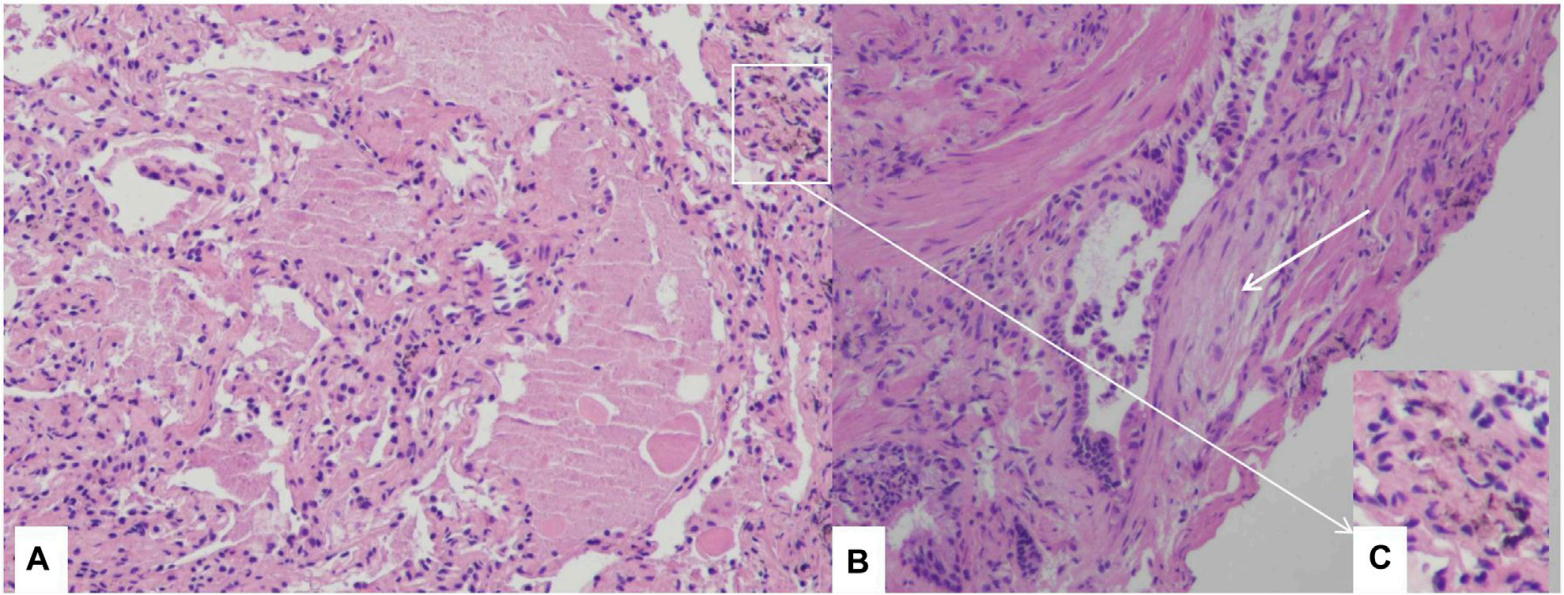
Histological examination through transbronchial lung cryobiopsy Section of lung biopsy specimen demonstrating the accumulation of amorphous eosinophilic materials in alveolar space, and alveolar septa are thickened **(A)**, H&E 10×). Fibroblast foci near the terminal bronchiole (white arrow) **(B)**, H&E 10×), dust particles in lung tissues **(C)**, H&E 10×).

Bronchoalveolar lavage (BAL) revealed infiltrating cell types consisting of 66% macrophages, 24% neutrophils, and 10% lymphocytes. However, there was no milky appearance in the BAL fluid. PAP was confirmed through transbronchial lung cryobiopsy of the lateral and posterior basal segment of the left lower lobe. Pathological examination revealed alveolar lumina filled with eosinophilic proteinaceous material, which stained pink with Periodic Acid Schiff (PAS) stain and tested positive for PAS, consistent with PAP. Fibroblast foci were also observed alongside the terminal bronchiole and there were a few dust particles in lung tissues. (Figure 2).

**FIGURE 3:**
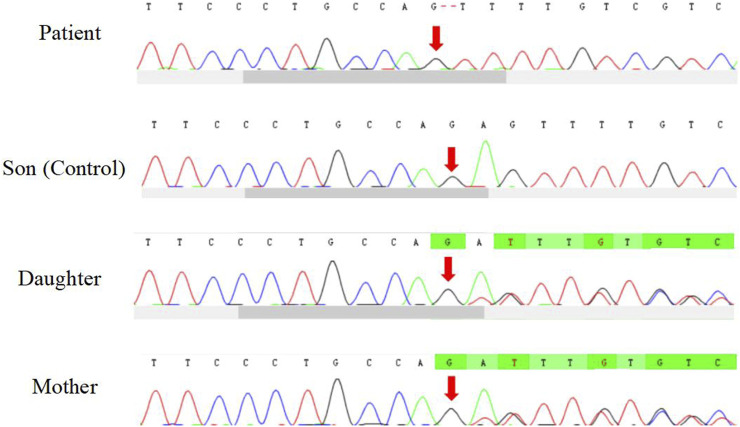
Homozygous frame shift mutation of CSF2RB.

**FIGURE 4:**
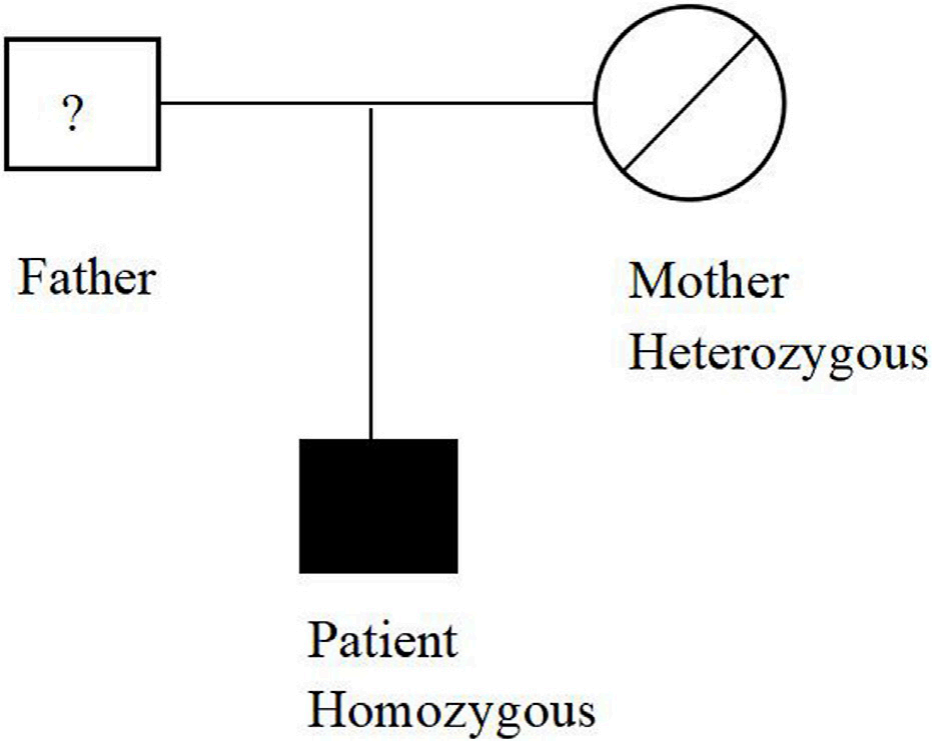
Distribution of the CSF2RB mutation in the studied family.

The patient underwent whole exome sequencing due to his parents being first cousins. The sequencing revealed a homozygous frame shift mutation (c.304_305del:p.S102Ffs*5) in exon 3 of the CSF2RB gene. This mutation is characterized by the loss of nucleotides at position 304–305 of the CSF2RB cDNA, resulting in the change of the 102nd codon of the mRNA from coding serine to phenylalanine. This led to the early generation of a termination codon at the 106th codon, causing protein truncation (Figure 3). The patient was found to be homozygous for this mutation, while his mother and sister were heterozygous carriers. However, his son did not carry the mutation. The patient’s father had passed away due to an unknown lung disease (Figure 4). This CSF2RB mutation impairs GM-CSF signaling, leading to PAP development by interfering with surfactant catabolism in alveolar macrophages in this patient.

## Discussion

HerPAP is a rare condition typically diagnosed in infancy but can also occur in adulthood. In this case, the initial manifestation was lung fibrosis, accompanied by symptoms of dyspnea and cough. The DLco test revealed severe impairment. Incidentally, a histopathological diagnosis of PAP was obtained through transbronchial lung cryobiopsy. Although the HRCT did not show typical PAP features, it displayed atypical features of fibrotic interstitial lung disease. Due to these atypical features, the pulmonary vein abnormality, and the consanguineous marriage of the patient’s parents, whole exome sequencing was performed, identifying CSF2RB mutation as a potential cause of PAP. While previous research suggested surfactant dysfunction as a cause of fibrotic lung disease (Luisetti et al., 2011). Occupational exposure to uncertainty dust may also contribute to the development of fibrotic interstitial lung disease in this patient. But the patients had no history of silica dust exposure. There were a few dust particles and had negative result of polarizing microscopes of the lung tissues. So there was no definite relationship of the dust inhalation with the fibrosis. It is also possible that the diffuse fibrotic lung disease represents the end-stage evolution of a previous PAP process. Nonetheless, fibrotic interstitial lung disease is a rare occurrence in hereditary PAP caused by CSF2RB mutations.

## Conclusion

Fibrotic interstitial lung disease is rare in PAP. Pathological tissues and whole exome sequencing are needed to aid in the final diagnosis.

## Data Availability

The datasets for this article are not publicly available due to concerns regarding participant/patient anonymity. Requests to access the datasets should be directed to the corresponding author.

## References

[B1] Borie R. Danel C. Debray M. P. Taille C. Dombret M. C. Aubier M. (2011). Pulmonary alveolar proteinosis. Eur. Respir. Rev. 20 (120), 98–107. 10.1183/09059180.00001311 21632797 PMC9487789

[B2] Luisetti M. Bruno P. Kadija Z. Suzuki T. Raffa S. Torrisi M. R. (2011). Relationship between diffuse pulmonary fibrosis, alveolar proteinosis, and granulocyte-macrophage colony stimulating factor autoantibodies. Respir. Care 56 (10), 1608–1610. 10.4187/respcare.01054 21513605

[B3] Tanaka T. Motoi N. Tsuchihashi Y. Tazawa R. Kaneko C. Nei T. (2011). Adult-onset hereditary pulmonary alveolar proteinosis caused by a single-base deletion in CSF2RB. J. Med. Genet. 48 (3), 205–209. 10.1136/jmg.2010.082586 21075760

[B4] Trapnell B. C. Nakata K. Bonella F. Campo I. Griese M. Hamilton J. (2019). Pulmonary alveolar proteinosis. Nat. Rev. Dis. Prim. 5 (1), 16. 10.1038/s41572-019-0066-3 30846703

